# Long-term bleeding events after mechanical aortic valve replacement in patients under the age of 60

**DOI:** 10.1007/s12471-014-0626-9

**Published:** 2014-11-19

**Authors:** B. M. Swinkels, B. A. de Mol, J. C. Kelder, F. E. Vermeulen, J. M. ten Berg

**Affiliations:** 1 Department of Cardiology, St. Antonius Hospital, PO Box 2500, 3435 CM Nieuwegein, the Netherlands; 2 Department of Cardiothoracic Surgery, Academic Medical Center, Amsterdam, the Netherlands; 3 Department of Cardiothoracic Surgery, St. Antonius Hospital, Nieuwegein, the Netherlands

**Keywords:** Aortic valve, Heart valve prosthesis, Intracranial haemorrhage, Coumarin

## Abstract

**Background:**

Although younger patients are supposed to be less susceptible to bleeding complications of mechanical aortic valve replacement (mAVR) than older patients, there is a relative paucity of data on this subject. Therefore, it remains uncertain whether younger patients are really at a lower risk of these complications than older patients.

**Methods:**

Incidence rates of bleeding events during 15 years of follow-up after mAVR were compared between 163 patients under 60 (group I), 122 patients between 60 and 65 (group II), and 145 patients over 65 (group III) years of age at operation. The target international normalised ratio (INR) was 3.0–4.0.

**Results:**

During 15 years of follow-up, the annual incidence rate of major bleeding events (excluding haemorrhagic stroke) was lower in the youngest as compared with the oldest group (3.0 versus 4.7 %, respectively; *p* = 0.030). However, the annual incidence rate of haemorrhagic stroke was as high in the youngest as in the two older groups (0.6 versus 0.7 % and 0.7 %, respectively; *p* = 0.928).

**Conclusions:**

With a target INR of 3.0–4.0, patients under 60 years of age are at equally high risk of haemorrhagic stroke after mAVR as older patients. This finding confirms the relevance of a lower target INR as used in international guidelines.

## Introduction

Mechanical aortic valve replacement (mAVR) is generally reserved for patients under the age of 60 years because of the durability of a mechanical prosthesis and a supposed lower susceptibility of younger patients to bleeding complications of oral anticoagulation therapy [1–5]. However, there is a relative paucity of data on long-term bleeding events after mAVR in patients under 60 years [6–8]. Therefore, it remains uncertain whether younger patients are really at lower risk of these complications than older patients. We aimed to compare incidence rates of bleeding events between patients under 60 and those over 60 years of age during 15 years of follow-up after mAVR.

## Methods

### Study design

In this retrospective longitudinal cohort study, 430 patients were followed for 15 years after mAVR, which was performed in the St. Antonius Hospital in Nieuwegein, the Netherlands, between 1990 and 1994. Incidence rates of bleeding events, occurring after discharge from hospital, were compared between three groups of patients: 163 patients under 60 (group I), 122 patients between 60 and 65 (group II), and 145 patients over 65 (group III) years of age at operation. Target international normalised ratio (INR) of oral anticoagulation therapy was 3.0–4.0, which was the standard at that time regarding mechanical aortic prostheses [9]. During follow-up, target INR did not change [10]. Data were obtained from our own or the referring cardiology departments, general practitioners, and telephone calls to patients and relatives. INR values within 48 h of the bleeding events, except the minor ones, were retrieved from the regional thrombosis services. The study object was agreed upon by the Hospital Committee on Ethics and Medical Experiments.

### Definitions

Bleeding events were divided into minor and major bleeding and haemorrhagic stroke events. Definitions were based on the official guidelines for reporting mortality and morbidity after cardiac valve interventions [11] and defined as follows. Minor bleeding: bleeding not requiring admission or blood transfusion. Major bleeding: fatal or nonfatal bleeding requiring admission or blood transfusion, excluding haemorrhagic stroke. Haemorrhagic stroke: focal neurological deficit of sudden onset as diagnosed by a neurologist, lasting more than 24 h and caused by cerebral bleeding.

### Data analysis

Calculation of late overall mortality was performed by Kaplan-Meier analysis. To calculate incidence rates of first bleeding events, Kaplan-Meier cumulative incidence rates were computed, whereas formal hypothesis testing was done by means of the log-rank test. To calculate incidence rates of multiple events (up to three per patient for minor or major bleeding events, and up to two for haemorrhagic stroke), linearised annual incidence rates (% per year, with exact 95 % confidence intervals [CI]) were computed, whereas formal hypothesis testing was done by means of an exact method.

## Results

### Baseline characteristics

Baseline characteristics are depicted in Table 1. The youngest patient was 21 and the oldest 80 years of age at operation. In all three groups more male than female patients were operated upon. In patients under 60 years at operation, mAVR was more often performed because of aortic regurgitation, as compared with aortic stenosis in the older groups. None of the patients had a history of haemorrhagic stroke.

**Table 1:** Baseline characteristics

	Group I (Age <60 y) *N* = 163	Group II (Age ≥60 ≤ 65 y) *N* = 122	Group III (Age >65 y) *N* = 145
Age (years)	50.4 ± 7.9	62.7 ± 1.8	68.7 ± 2.7
Male	117 (71.8)	77 (63.1)	95 (65.5)
EuroSCORE II	1.2 ± 1.1	1.9 ± 1.9	2.6 ± 3.0
Logistic EuroSCORE	2.5 ± 2.2	3.6 ± 4.2	5.6 ± 7.1
STS score (version 2.73)	1.0 ± 0.9	1.5 ± 1.2	2.0 ± 1.7
Concomitant CABG	26 (16.0)	32 (26.2)	48 (33.1)
Predominant aortic stenosis	108 (66.3)	104 (85.2)	120 (82.8)
Body mass index (kg/m^2^)	26 ± 3	26 ± 4	25 ± 3
Insulin dependent diabetes	0	1 (0.8)	5 (3.4)
Non-insulin dependent diabetes	5 (3.1)	7 (5.7)	18 (12.4)
Paroxysmal atrial fibrillation	9 (5.5)	9 (7.4)	17 (11.7)
Permanent atrial fibrillation	4 (2.5)	2 (1.6)	14 (9.7)
Renal failure*	3 (1.8)	0	2 (1.4)
Hypertension	76 (46.6)	58 (47.5)	75 (51.7)
History of ischaemic stroke	6 (3.7)	1 (0.8)	13 (9.0)
History of gastrointestinal bleeding	0	5 (4.1)	7 (4.8)

Values are presented as mean ± standard deviation or *N* (%)

*CABG* coronary artery bypass grafting

*Serum creatinine >2.3 mg/dl (200 μmol), including dialysis

### Mechanical protheses

The different types of implanted mechanical aortic valve prostheses are depicted in Table 2. They were similarly distributed among the three groups, half of them being bileaflet and the other half tilting disc prostheses.

**Table 2:** Implanted mechanical aortic valve prostheses

	Group I (Age <60 y) *N* = 163	Group II (Age ≥60 ≤ 65 y) *N* = 122	Group III (Age >65 y) *N* = 145
Bileaflet prostheses			
St. Jude Medical	79 (48.5)	58 (47.5)	68 (46.9)
St. Jude Medical Hemodynamic Plus	1 (0.6)	1 (0.8)	1 (0.7)
Sorin Bicarbon	3 (1.8)	4 (3.3)	4 (2.8)
Tilting disc prostheses			
Sorin Allcarbon	80 (49.1)	59 (48.4)	72 (49.7)

Values are presented as *N* (%)

### Follow-up

Mean follow-up after mAVR was 18.1 ± 1.2 years. All patients were followed for at least 15 years after operation or until death. Follow-up was complete in all patients.

### Thirty-day and late mortality

Thirty-day mortality rates were 1.2, 1.6, and 2.8 % in group I, II, and III, respectively. Kaplan-Meier late overall cumulative mortality rates (including the patients who died within 30 days of operation) at 15 years of follow-up were 27.6 % (95 % CI: 20.4–34.2), 53.6 % (95 % CI: 43.8–61.7), and 73.1 % (95 % CI: 64.8–79.4) in group I, II, and III, respectively.

### Bleeding events

Incidence rates of bleeding events are depicted in Table 3. Total numbers of patient-years of follow-up were 2479, 1541, and 1481 years in group I, II, and III, respectively. During 15 years of follow-up, the annual incidence rate of major bleeding events (excluding haemorrhagic stroke) was lower in the youngest as compared with the oldest group (3.0 versus 4.7 %, respectively; *p* = 0.030). However, the annual incidence rate of haemorrhagic stroke was as high in the youngest as in the two older groups (0.6 versus 0.7 % and 0.7 %, respectively; *p* = 0.928). Incidence rates of bleeding events did not change over the 15 years of follow-up.

**Table 3:** Cumulative and annual incidence rates of bleeding events

	Group I (Age <60 y) *N* = 163	Group II (Age ≥60 ≤ 65 y) *N* = 122	Group III (Age >65 y) *N* = 145	*P*- value
Minor bleeding				
Kaplan-Meier cumulative incidence of first events (%; 95 % CI)	45.7 (36.9–53.3)	47.9 (36.6–57.1)	51.3 (38.8–61.3)	*P* = 0.783
Linearised annual incidence rate of first or recurrent events (%; 95 % CI)	5.5 (4.6–6.5)	6.2 (5.1–7.6)	7.0 (5.7–8.4)	*P* = 0.278
Major bleeding (excluding haemorrhagic stroke)				
Kaplan-Meier cumulative incidence of first events (%; 95 % CI)	29.9 (21.9–37.1)	34.2 (23.7–43.3)	42.7 (31.2–52.3)	*P* = 0.052
Linearised annual incidence rate of first or recurrent events (%; 95 % CI)	3.0 (2.4–3.7)	4.0 (3.1–5.1)	4.7 (3.7–5.9)	*P* = 0.030*
Haemorrhagic stroke				
Kaplan-Meier cumulative incidence of first events (%; 95 % CI)	7.6 (3.2–11.9)	9.6 (3.3–15.6)	7.5 (2.6–12.1)	*P* = 0.947
Linearised annual incidence rate of first or recurrent events (%; 95 % CI)	0.6 (0.3–1.0)	0.7 (0.4–1.3)	0.7 (0.3–1.2)	*P* = 0.928

*CI* confidence interval

*Group I vs. III

### Related INR values

During 15 years of follow-up, a total of 206 first or recurrent major bleeding events (excluding haemorrhagic stroke) and 36 first or recurrent haemorrhagic stroke events occurred. The mean INR within 48 h of these 242 events was 4.0 ± 1.5 (range: 1.5–9.4). In 74 (30.6 %) of these 242 events, the INR value was not available.

### Related INR values in patients under 60 years at operation

In the 163 patients under 60 years of age at operation (group I), 48 patients suffered a first major bleeding event (excluding haemorrhagic stroke). The related mean INR was 4.1 ± 1.2 (range: 2.3–7.5), whereas the INR was unavailable in 15 (31.3 %) of these patients. In the 48 patients with a first major bleeding event (excluding haemorrhagic stroke), one event was fatal (related INR: 4.2). In group I, 14 patients suffered a first haemorrhagic stroke. The related mean INR was 3.4 ± 0.8 (range: 2.2–4.8), whereas the INR was unavailable in 3 (21.4 %) of these patients. In the 14 patients with a first haemorrhagic stroke, 8 events were fatal. The related mean INR was 3.8 ± 0.7 (range: 3.0–4.8), whereas the INR was unavailable in 2 (25.0 %) of these patients.

In group I, 15 patients were younger than 40 years at operation. Three of them, all males, suffered a first major bleeding event (excluding haemorrhagic stroke) at age 34, 29, and 46 years (5.8, 0.8, and 8.2 years after mAVR, respectively). None of these events were fatal and the related INR values were 3.2, 3.8, and 4.1, respectively. One of these 15 patients suffered a haemorrhagic stroke at age 44 (10 years after mAVR). This event was fatal (related INR: 3.4).

## Discussion

In this study, incidence rates of bleeding events during 15 years of follow-up after mAVR were determined in a group of patients under 60 years of age, and compared with two groups of patients aged between 60 and 65 and over 65 years at operation, respectively. The patients under 60 years of age were not at low risk of long-term bleeding complications as compared with the older patients. Although the annual incidence rate of major bleeding events (excluding haemorrhagic stroke) was lower in the youngest as compared with the oldest group (3.0 versus 4.7 %, respectively; *p* = 0.030), the annual incidence rate of haemorrhagic stroke was as high in the youngest as in the two older groups (0.6 versus 0.7 % and 0.7 %, respectively; *p* = 0.928). We do not know why the patients under 60 years, despite their younger age, were at equally high risk of haemorrhagic stroke as the older patients. There were no suggestions of a selection of younger patients more prone to bleeding, because risk factors for bleeding (female gender, renal failure, hypertension, history of ischaemic stroke or gastrointestinal bleeding) were not more common in the youngest than in the older groups (Table 1). Our finding that younger patients on oral anticoagulation therapy were not at lower risk of haemorrhagic stroke than older patients is confirmed by a study in 42 both younger and older patients (24 % of patients under 65, 59 % of patients between 65 and 79, and 17 % of patients over 79 years of age) who suffered a haemorrhagic stroke while they were on oral anticoagulation therapy because of atrial fibrillation (57 % of patients), venous thromboembolism (24 % of patients), and/or prosthetic heart valves (14 % of patients) [12]. The mean INR on admission in this study was 3.6 ± 2.1 for the whole patient group (INR values in the patients under the age of 65 were not available), which is comparable with the mean INR of 3.4 ± 0.8 within 48 h of the haemorrhagic stroke events in the youngest age group of the present study. The target INR of 3.0–4.0 in our patients did not change during the 15 years of follow-up. From the literature it is known that annual incidence rates of major bleeding and haemorrhagic stroke events in patients on oral anticoagulation therapy with a target INR of 3.0–4.0 are approximately 3.0 and 0.6 %, respectively (comparable with the present study), while annual incidence rates of major bleeding and haemorrhagic stroke events with a lower target INR of 2.0–3.0 are reported to be approximately 2.0 and 0.5 %, respectively [13–17]. Excessive INR values (above 5.0) are associated with very high bleeding rates [18, 19]. Current [20, 21] and past [22] international guidelines recommend a target INR of 2.0–3.0 to 2.5–3.5 (depending on prosthesis thrombogenicity and patient-related risk factors for thromboembolism) for most mechanical aortic valves of the last decades, including the prostheses used in the present study. These target INR values are based on studies, including three major randomised trials [23–25], weighing thromboembolic risks against haemorrhagic risks in patients on oral anticoagulation therapy after mechanical heart valve replacement. Although we do not know what the bleeding figures in our patients would have been if the target INR had been as low as in the international guidelines, it seems plausible that with a lower target INR the incidence rates of both major bleeding and haemorrhagic stroke events would have been lower in all three age groups. An important shortcoming of the present study is the high percentage (30.6 %) of unavailable INR values within 48 h of the major bleeding and haemorrhagic stroke events. It is therefore not known how many patients might have suffered these events due to an excessively high INR. However, the INR values which were available within 48 h of the major bleeding or haemorrhagic stroke events were not excessively high.

## Conclusions

With a target INR of 3.0–4.0, patients under 60 years of age are at equally high risk of haemorrhagic stroke after mAVR as older patients. This finding confirms the relevance of a lower target INR as used in international guidelines.
